# Elucidating the enigmatic biology of arteriviruses through receptor discovery

**DOI:** 10.1128/jvi.01787-25

**Published:** 2026-03-30

**Authors:** Adam L. Bailey

**Affiliations:** 1 University of Wisconsin–Madison 5228, Madison, Wisconsin, USA; Indiana University Bloomington, Bloomington, Indiana, USA

**Keywords:** arterivirus, lactate dehydrogenase elevating virus, porcine respiratory and reproductive syndrome virus, equine arteritis virus, simian hemorrhagic fever virus, simian arterivirus, simartevirus, arteriviridae, nidovirales

## Abstract

Arteriviruses are diverse +ssRNA viruses (*Nidovirales* Order; *Arteriviridae* Family) that infect a variety of mammals. Arterivirus infections can manifest in a variety of ways, ranging from viral hemorrhagic fever to persistent sub-clinical infection. A perplexing feature of arterivirus biology is the unusual network of small glycoproteins that decorate the virion surface. How these glycoproteins mediate viral entry into target cells remains poorly understood, but it is widely accepted that the arterivirus entry process is novel. This review highlights recent advances in the characterization of arterivirus entry receptors and examines unique features of arterivirus biology—including disease, persistence, tropism, evolution, and cross-species transmission—through the lens of receptor utilization.

## INTRODUCTION

### Arteriviruses are understudied mammalian viruses

**Fig 1:**
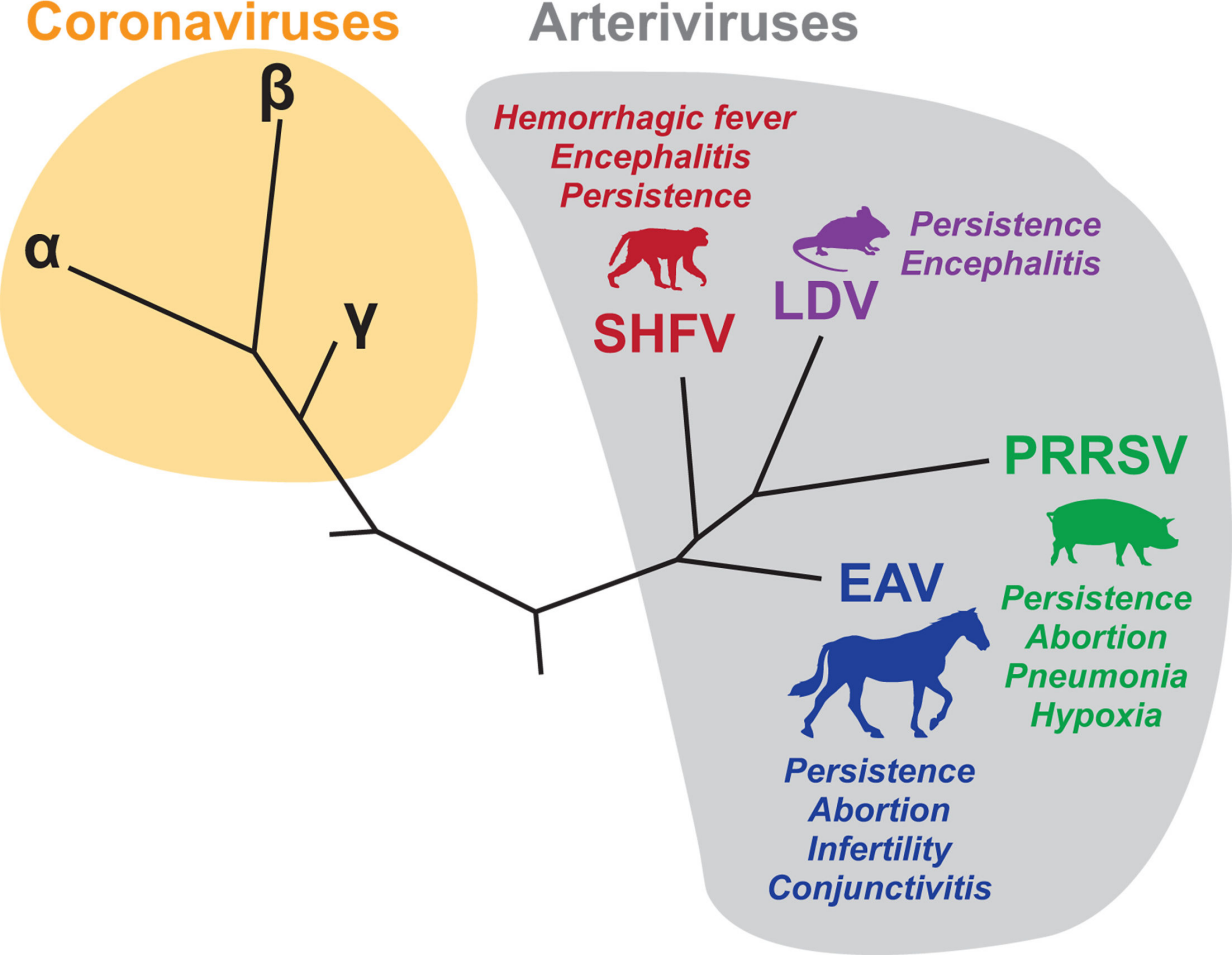
The *Arteriviridae* family. Simplified phylogeny showing the four major arteriviruses in relation to the *Coronaviridae* family within the *Nidovirales* order. Disease manifestations are shown next to the silhouettes of arterivirus hosts. Note that many of the newly discovered viruses in both the *Arteriviridae* and *Coronaviridae* families are not depicted for the sake of simplicity.

Arteriviruses are enveloped +ssRNA viruses related to Coronaviruses (both are in the Order *Nidovirales*) (1, 2). The natural diversity of the *Arteriviridae* family is vast, with host-vs-virus phylogenetic comparisons revealing multiple historical cross-species transmission events (3, 4). The four arteriviruses most studied in the laboratory serve as foundational “prototypes” from which most knowledge about the *Arteriviridae* family has been generalized: equine arteritis virus (EAV), porcine reproductive and respiratory syndrome virus (PRRSV), simian hemorrhagic fever virus (SHFV), and lactate dehydrogenase-elevating virus (LDV) (Fig. 1). Transmission of these viruses can occur via multiple routes (respiratory droplet, sexual, vertical, and blood-borne), and infections can cause a range of diseases, including fatal pneumonia, abortion, encephalitis, and hemorrhagic fever (1). Perplexingly, some arteriviruses can also cause life-long infection without inducing overt disease, despite high virus titers in the blood (5–7). Macrophages are thought to be the primary target of arterivirus infection *in vivo*, though some arteriviruses, such as EAV, have a broader cellular tropism (1). Importantly, the relationship between macrophage tropism, persistence, and disease remains poorly understood.

### Arteriviruses have a history of cross-species transmission and emergence

The history of two arteriviruses highlights the potential of these viruses to infect new hosts and cause disease.

SHFV was discovered in 1964 following an outbreak of lethal hemorrhagic fever in several species of Asian-origin macaque monkeys at a primate facility in Bethesda, MD, USA (8, 9). Since this time, SHFV has sporadically caused disease outbreaks in captive macaque colonies (10, 11). Although African-origin monkeys were long suspected to be the source of these outbreaks, it was not until recently that highly divergent SHFV-like viruses—collectively called “simian arteriviruses”—were discovered infecting wild monkeys in Africa. Subsequent investigations showed that simian arteriviruses were surprisingly widespread in monkeys throughout sub-Saharan Africa (12, 13). This prompted a reexamination of historical “SHFV” outbreaks using modern sequencing techniques, which revealed that several outbreaks were actually caused by unique simian arteriviruses that were only ~50% identical at the nucleotide level (14), highlighting the collective potential of simian arteriviruses to transmit between different primate species (15). Prospective infection studies of captive primates with simian arteriviruses have shown that the disease caused by these infections is highly variable and unpredictable, ranging from persistent subclinical infection to fatal hemorrhagic fever in monkeys of both African and Asian origin (7, 16, 17).

In 1991, PRRSV was discovered as the causative agent of a “mystery swine disease” that first emerged in the United States in 1987 (18). By 1990, the disease had become widespread in North America and began appearing in pig farms across Europe. Curiously, sequence analysis showed that European PRRSV and North American PRRSV shared only ~60% nucleotide identity, prompting their separation into PRRSV-1 and PRRSV-2 species, respectively. The genetic distance between PRRSV-1 and PRRSV-2 suggests independent sources for these co-occurring epizootics (18). Though the natural host and origin of these viruses remains unknown, phylogenetic analysis implicates rodents as a likely source (19). Transmission of PRRSV occurs predominantly via the airborne/respiratory route. PRRSV typically causes only acute infection and does not persist in individuals like some other arteriviruses; however, the virus moves slowly through herds infecting only a small proportion of individuals at any given time, resulting in herd-level persistence. This has stymied control, eradication, and prevention efforts. Porcine reproductive and respiratory syndrome (PRRS), the disease caused by PRRSV infection, is highly variable in adult pigs, with many pigs showing mild or no overt signs of disease. More severe cases present as an influenza-like illness that can progress to hypoxic respiratory failure and predispose the animal to secondary bacterial pneumonia. In the past decade, China has seen the emergence of “highly pathogenic” PRRSV variants that can kill ~20% of adult pigs (20, 21). However, the greatest economic damage results from PRRSV infection of piglets during the perinatal period. PRRSV efficiently transmits across the placenta in pregnant sows, often resulting in herd-wide “abortion storms” and stillbirths. Infection of neonatal piglets can also impact growth and development, ultimately resulting in “under-finished” adults of low value. Thus, PRRSV causes billions of US dollars in economic losses annually and adds to the growing problem of food insecurity (22).

### Arterivirus persistence is poorly understood

Arteriviruses are adept at evading host immune responses, with many arteriviruses causing life-long high-titer viremia in their natural host (6, 14, 15). Although how arteriviruses accomplish this feat remains poorly understood, it is widely acknowledged that the mechanism of persistence employed by arteriviruses is novel (5, 23). Indeed, the mouse arterivirus LDV was a popular model of “failed immunity” in the 1960-1990s, when it was shown that LDV infection alters several immune system functions (24). However, longstanding failure to develop an *in vitro* culture system for LDV ultimately caused it to fall into obscurity by the late 1990s.

### The arterivirus glycoproteins are highly unusual

**Fig 2:**
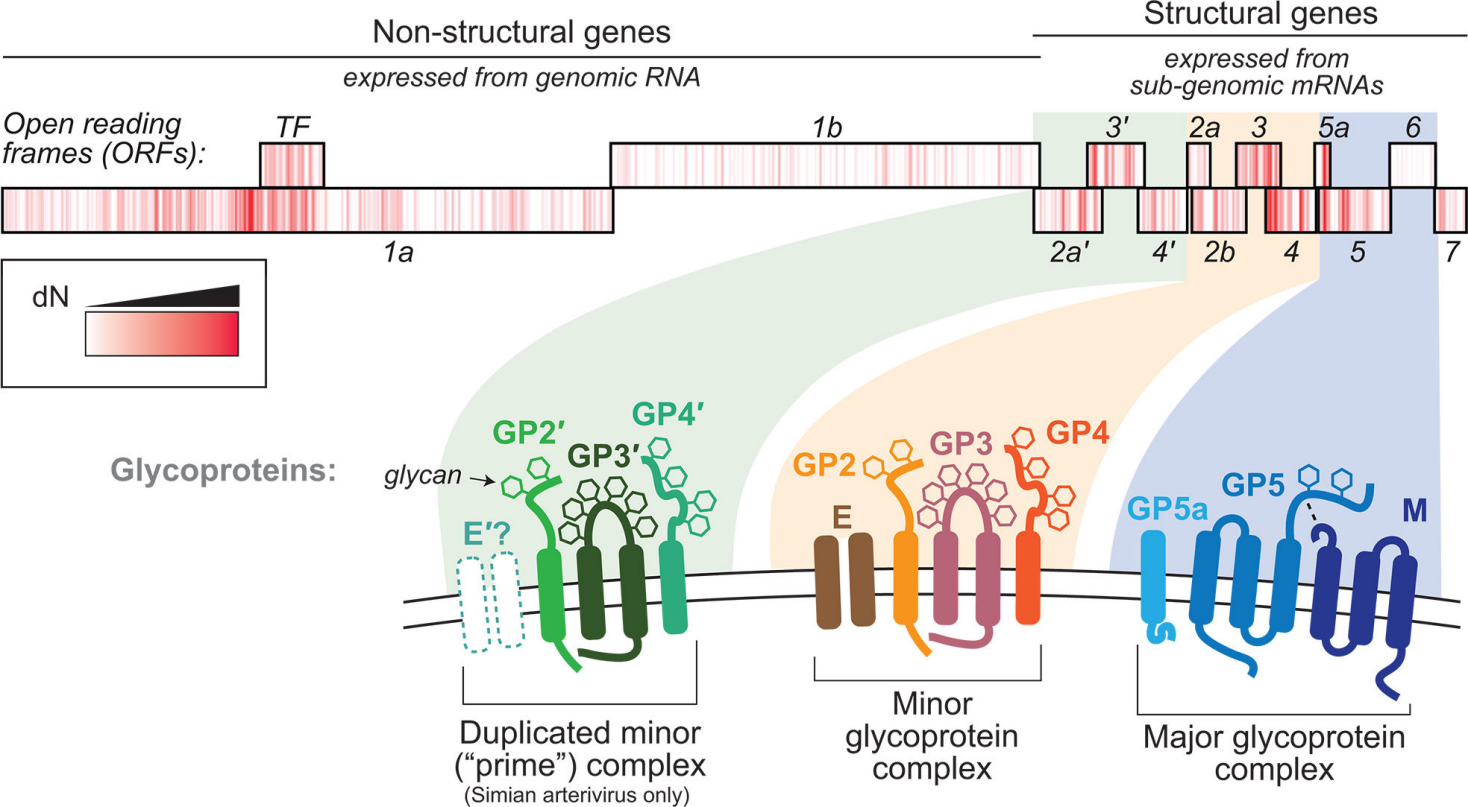
The arterivirus envelope glycoproteins. The arterivirus glycoproteins are encoded by a series of overlapping open reading frames (ORFs) that are located in the 3′ of the genome. These structural genes are expressed via subgenomic mRNAs via a complex and highly regulated process. Shown is a schematic of the ORFs from Drakensberg Mountain vervet virus (DMVV-1), which encodes the duplicated minor complex (i.e., “prime complex”) characteristic of all simian arteriviruses. Note that DMVV-1 does not encode an ORF2b′, which appears to be variably encoded by simian arteriviruses and is the only glycoprotein dispensable for SHFV infectivity (28). Overlaid on each ORF is a heatmap of the non-synonymous substitution rate (dN, generated using the SNAP server [32]), that highlights regions potentially involved in immune escape. Below, cartoons depict the topology of each glycoprotein, in the order with which they are encoded in the genome. The “major glycoprotein complex” is most abundant and consists of a disulfide-linked heterodimer between GP5 and M (dashed line). The “minor glycoprotein complex” consists of a GP2/3/4 heterotrimer that also forms interactions with E, which is thought to be an ion channel. The duplicated “prime complex” in the simian arteriviruses is also shown.

In contrast to many RNA viruses that have one or two glycoproteins (e.g., spike) on their surface, arteriviruses have a complex network of 7–11 unique glycoproteins decorating their surface, many of which have multiple transmembrane domains and unusually small ectodomains (Fig. 2) (25, 26). These glycoproteins are thought to coalesce into “major” and “minor” complexes, which are composed of GP5:M heterodimers and GP2:3:4 heterotrimers, respectively, with the “E” protein affiliating loosely with the minor complex. The simian arteriviruses (including SHFV) all contain an additional four genes that encode a putative second minor glycoprotein complex (denoted by the prime [′] symbol)—the host factor(s) engaged by this second minor complex remain completely undefined (12, 27). All arterivirus structural proteins are required for production of infectious virus (with the possible exception of simian arterivirus protein 2b′), implying a coordinated entry process that involves cooperation between the glycoproteins (28). Many of the glycoproteins have large intrinsically disordered regions, and none of the glycoproteins resemble a typical class I, II, III, or IV viral fusion protein (29). These unique attributes have hindered investigations of arterivirus glycoproteins by standard biochemical or electron microscopy techniques, resulting in a dearth of information for building accurate biochemical structure models. *In silico* modeling of these glycoproteins is in the early stages, but holds promise for revealing novel insights into the higher-order interactions between the glycoproteins (30, 31). All together, the unusual features of these glycoproteins imply a novel and complex mechanism of viral entry.

### Limitations in understanding the diversity of arteriviruses and their receptors

Arteriviruses have mainly been discovered in two ways: the identification of a disease-causing agent or unbiased metagenomic sequencing of samples from randomly sampled animals. This latter approach has demonstrated that many arterivirus infections show little or no signs of disease in “natural hosts,” suggesting that our current understanding of arterivirus diversity may be a significant underestimate (3, 19, 33–35). While many arteriviruses likely remain to be discovered, several technical barriers (e.g., sequence divergence and lack of serological tools) limit further arterivirus discovery. Additionally, the diversity of tissues and anatomic locations in which arteriviruses can be found (e.g., blood, respiratory, and semen) further muddles discovery efforts, especially in endangered or protected animals. 

Beyond viral discovery, there are currently several limitations to studying divergent arteriviruses in the laboratory. For one, infectious biological material is often not acquired during sequencing-based discovery approaches. In rare circumstances where biological material is available, many divergent arteriviruses will not infect common immortalized cell lines, such as MA-104, for unknown reasons. Although several reverse genetics systems provide a blueprint for the synthesis of arteriviruses discovered using metagenomic sequencing, the lack of host systems for rescue and cultivation of divergent viruses remains a major limitation.

In terms of receptor discovery, the arterivirus glycoproteins also present unique challenges. The sheer number of unique glycoproteins—and their assembly in the endoplasmic reticulum/Golgi complex—has precluded the development of lentiviral or vesicular stomatitis virus (VSV)-based pseudovirus systems, which classically bud from the plasma membrane. Additionally, the arrangement of arterivirus glycoproteins in heteromeric complexes, a poor understanding of major and minor complex assembly, cohesion, and conformational dynamics during entry, and the relatively small ectodomains of these glycoproteins have limited interrogation by biochemical methods. Thus, a confluence of factors has stymied our understanding of arterivirus:receptor interactions.

### Current understanding of arterivirus entry factors: a PRRSV-centric perspective

Most arterivirus research over the past several decades has focused on PRRSV due to its economic impact on the pig farming industry. In 2007, a screen using cDNA from porcine alveolar macrophages showed that porcine CD163 could render cells susceptible to PRRSV infection (36). CD163 functions physiologically as a receptor for hemoglobin-haptoglobin complexes, scavenging these from circulation. CD163 expression is restricted to macrophages, with limited expression on other myeloid lineages (e.g., some monocytes and dendritic cells) that varies by species, tissue compartment, and cellular activation state. Since the initial discovery of a role for CD163 in PRRSV infection, numerous studies have shown that CD163 is necessary and often sufficient for PRRSV infection of cells in culture, as summarized in reference 37. Classically, the structure of CD163 has been conceptualized as linear, with nine cysteine-rich scavenger receptor (SRCR) domains coalescing into “beads along a string.” Within this context, genetic and biochemical studies of porcine CD163 have demonstrated an important role for SRCR domain 5 (38–42) in PRRSV entry.

However, more recent data show that CD163 switches between a monomeric “inactive” and a trimeric “active” form that binds hemoglobin/haptoglobin at the cell surface (43), and it is becoming increasingly clear that the beads-on-a-string model is an oversimplification that does not incorporate higher-order structural features of CD163. Consistent with this notion, more comprehensive studies of PRRSV have revealed interactions with several SRCR domains on CD163 as well as the membrane-proximal proline-serine-threonine (PST) domain II (44). Direct visualization of the entire CD163 molecule bound to PRRSV has so far not been possible, though this remains an important goal in the field. Multiple PRRSV glycoproteins have been shown to interact with CD163. Though the precise receptor-binding domain (RBD) remains to be fully elucidated, studies consistently point to proteins of the minor complex as CD163’s binding partners (31, 40, 45). Perhaps the most compelling data for the importance of CD163 in the PRRSV lifecycle has come from pigs genetically modified to lack CD163, the SRCR5 domain, or the PST-II domain, all of which are highly resistant to PRRSV infection (46–52).

In addition to CD163, a plethora of host molecules have been shown to enhance PRRSV internalization *in vitro*. The most well-characterized of these additional factors is Siglec-1 (aka, sialoadhesin, CD169), a sialic-acid-binding protein (i.e., “sialoadhesin”) that is predominantly expressed on macrophages in secondary lymphoid organs (53). Siglec-1 enhances internalization of several sialylated bacteria and viruses, including PRRSV, in certain experimental systems (54–60). However, Siglec-1 is neither necessary nor sufficient for PRRSV infection, and pigs engineered to lack Siglec-1 are fully PRRSV-susceptible (61). More recently, the non-muscle myosin heavy chain 9 (MYH9) protein has been implicated in PRRSV entry, with MYH9 aggregation via interactions with the GP5 (major complex) seeming to play a role in PRRSV internalization (62–66). Interestingly, piglets administered blebbistatin—a relatively toxic inhibitor of myosin II—appeared to be protected from PRRSV (62), but a role for MYH9 in PRRSV infection *in vivo* needs further investigation. Additional host factors, including vimentin, CD151, DC-SIGN, and heparan sulfate, have been implicated as pro-viral factors in the PRRSV life cycle; however, the precise role of these host factors in PRRSV entry, their *in vivo* relevance, and their generalizability to other arteriviruses remains largely unclear (37).

## RECENT ADVANCES

### CD163 utilization is not unique to PRRSV

While CD163 is essential for PRRSV infection, its role in the entry of other arteriviruses has only recently been investigated. Following up on a key observation that anti-CD163 antibodies could block SHFV infection of MA-104 cells (67), Warren et al. showed that CD163 was essential for SHFV entry (68). They also showed that the human CD163 ortholog was fully supportive of SHFV entry, and that certain human cell lines were susceptible to infection when human CD163 was ectopically expressed, indicating that the entire SHFV infection cycle is molecularly compatible with a human host. Interestingly, they also showed that human monocyte-derived macrophages are not supportive of SHFV infection. This discrepancy remains unexplained but may be due to differences between tissue-resident macrophages and monocyte-derived macrophages or the expression of a restriction factor that is absent in susceptible immortalized human cell lines.

### CD163 is also required for LDV entry

Inspired by the notion that CD163 could act as a receptor for multiple divergent arteriviruses, we recently demonstrated that CD163-knockout mice are completely protected from LDV infection. Interestingly, immortalized murine macrophage cell lines do not express CD163, which may be due to the fact that most of these cell lines are not derived from tissue-resident macrophages. Only tissue-resident macrophages—and not monocytes or monocyte-derived macrophages—express CD163 in mice. This CD163 expression pattern differs from that of other mammals and has only recently been recognized (69). However, ectopic expression of murine CD163 rendered multiple cell lines highly susceptible to LDV infection (70). Thus, the longstanding inability to culture LDV *in vitro* was due to a lack of the critical viral receptor CD163.

### CD81 is a putative receptor for EAV

MA-104 cells are an immortalized line derived from African green monkey (*Chlorocebus sabaeus*) kidney epithelium that aberrantly expresses CD163. These cells are also unusual in their ability to support infection with PRRSV, SHFV, and EAV. We used MA-104 cells to perform genome-wide CRISPR-knockout screens with the goal of identifying host-dependency factors that were conserved or unique among each of these three viruses. Following PRRSV and SHFV infection, surviving MA-104 cells were enriched for sgRNAs targeting several genes including CD163, validating this approach (71). Interestingly, CD163-targeting sgRNAs were not significantly enriched in cells that survived EAV infection; instead, the multifunctional tetraspanin CD81 appeared as a top hit in the EAV survivors. CD81 sgRNA enrichment was not observed in either the SHFV or PRRSV screens, suggesting that CD81 and CD163 play functionally equivalent roles in the arterivirus entry process. Although this hypothesis remains to be fully proven, we have so far shown that CD81 knockout renders various cell lines completely resistant to EAV infection. However, EAV can be produced from CD81-knockout cells via transfection of EAV genetic material (i.e., “entry bypass”), implicating CD81 in EAV entry. Additionally, EAV infectivity can be “blocked” via pre-incubation of EAV with soluble CD81. To examine whether CD81 can serve as a species barrier to EAV infection, we cloned/synthesized CD81 orthologs from known arterivirus hosts. This identified CD81 from the common brushtail possum as unsupportive of EAV infection, allowing us to map this resistance to the “D” alpha-helix on CD81’s large extracellular loop (LEL) by testing and creating various possum/horse chimeric CD81s. Thus, it appears that the apical loop of CD81 is essential for EAV engagement and that sequence variation in CD81 can serve as a molecular barrier to cross-species infection (72).

### The neonatal Fc receptor (FcRn) is a pan-arterivirus receptor

Beyond CD163 (for SHFV and PRRSV) and CD81 (for EAV), the MA-104 CRISPR screen also identified the neonatal Fc receptor (gene: *FCGRT*; protein: FcRn) and beta-2 microglobulin (gene: *B2M*; protein: β2m) as critical for all three arteriviruses. FcRn plays an important role in the transport and homeostatic maintenance of IgG and albumin throughout the body, and its functioning depends upon complexing with β2m; thus, these hits were viewed as interconnected. Similar to our analysis of CD81’s role in EAV infection, we explored the role of FcRn/β2m in arterivirus entry by showing that multiple different FcRn-knockout cell lines are completely resistant to arterivirus infection even while CD163 is overexpressed, indicating that neither FcRn nor CD163 (or CD81) is sufficient to mediate arterivirus infection by itself. The resistance imparted by FcRn-knockout can be “bypassed” via transfection of viral genomic material, and arterivirus infection can be blocked via pre-incubation of cells with anti-FcRn antibodies. FcRn overexpression greatly enhances the susceptibility of multiple cell lines to arterivirus infection, resulting in more accurate quantitation of infectious virus (e.g., via plaque assay) and the successful propagation of previously uncultivable simian arteriviruses. Finally, we demonstrated that FcRn sequence differences among various arterivirus hosts can serve as a molecular barrier to cross-species infection (71).

## OUTSTANDING ENIGMAS IN ARTERIVIRUS RECEPTOR BIOLOGY

### Mechanism(s) of arterivirus entry

Several studies have characterized arterivirus entry using chemical inhibitors (67, 73–75). Together, this research suggests that arteriviruses enter cells via clathrin/dynamin-mediated endocytosis, require a low pH for membrane fusion, and may rely on the enzymatic activity of specific endosomal cathepsins (e.g., cathepsin E). Although some of these requirements have been evaluated in porcine alveolar macrophages, an important caveat to these studies is the use of immortalized cell lines with aberrant receptor expression (i.e., MA-104 and MARC-145). Nevertheless, the field is now well positioned to start linking these biochemical aspects of arterivirus entry to the timing and location of arterivirus receptor utilization. Many enveloped viruses that rely on multiple host factors for entry (e.g., filoviruses, Lassa virus, and hepatitis C virus) do so in a stepwise process: first, engagement of host factors at the cell surface mediates internalization of the virion into the endosome (internalization receptors); then, engagement of host factors in the endosome facilitates the fusion of viral and host membranes (fusogenic receptors) (2). The reliance of arteriviruses on multiple non-redundant host factors suggests that these viruses also progress through a stepwise entry process. It also follows that, given the organization of arterivirus glycoproteins into distinct complexes, specific steps in the entry process (i.e., internalization and fusion) may be orchestrated by distinct complexes (i.e., major and minor). Considering the critical importance of CD163 for productive LDV, PRRSV, and SHFV infection, it has long been assumed that CD163 is the fusogenic arterivirus receptor. However, this has not been definitively proven, and some preliminary evidence suggests that FcRn may play the role of fusogen in arterivirus entry (76). While both CD163 and FcRn could play the role of fusogenic receptor, it seems more likely that arterivirus entry is a multi-step process in which internalization is driven by interactions between the major complex and FcRn or Siglec-1, followed by a membrane fusion step that is mediated by minor complex interactions with CD163 (or CD81 for EAV) in the acidified endosome. Given the large number of variables at play (Table 1), this model may be overly simplistic, out-of-order, improperly mixed-and-matched, or otherwise incorrect. As we learn more about the arterivirus entry process, we must keep an open mind; the unusual features of the arterivirus glycoproteins raise the possibility that arterivirus entry mechanisms are substantially different from those other known viruses.

**TABLE 1:** Factors implicated in arterivirus entry to date

Host factors	Viral factors
CD163	Major complex
CD81 (EAV only?)	M
Siglec-1 (CD169)	GP5
FcRn	GP5a
β2m	Minor complex
Albumin?	GP2
IgG?	GP3
Sialic acid	GP4
CXCL16?	E
Heparan sulfate	Prime complex (simian only)
DC-SIGN	GP2a′
Vimentin	GP2b′
CD151	GP3′
MYH9	GP4′
HSPA8	
TIM-1,4	
Siglec-10	
Syndecan-4	

### Relationship between receptor utilization and cellular tropism

At the cellular level, the display of receptors compatible with viral entry is a prerequisite for productive infection. The cells targeted for infection—their identity, the physiologic dysfunction brought about by their destruction, and their role in the immune response—also play a central role in the disease process. Thus, the pattern of receptor display should have a clear mechanistic link to disease. Why then do different arteriviruses cause such vastly different diseases?

A logical extension of the entry model proposed above is that successful arterivirus entry depends upon the proper display of *both* internalization receptors and fusogenic receptors. Thus, variation in expression (or sub-cellular compartmentalization) of these receptors could result in significant differences in the cell types susceptible to arterivirus infection. This is most clearly illustrated by EAV, which displays a unique and “expanded” tissue tropism compared to other arteriviruses that is almost certainly the result of the broader expression profile of CD81 compared to CD163. However, it is likely that more nuanced aspects of arterivirus:receptor interactions ultimately result in significant differences in viral tropism that have important implications for arterivirus disease, persistence, and cross-species transmission. For example, the “efficiency” with which arteriviruses interact with the suite of available receptors may be virus- and host-specific, with some viruses favoring the use of certain internalization receptors over others. Receptor density likely impacts this preference, with high receptor expression/density compensating for minor inefficiencies by providing more favorable stoichiometry (71). It is also entirely possible that receptor expression/utilization changes throughout the course of infection, with distinct combinations of receptors and cell types supporting acute versus sub-acute versus chronic phases of infection. Host-specific differences in receptor expression are also likely important. In mice, for example, monocytes do not express CD163 and do not upregulate CD163 when they differentiate into macrophages—a distinct departure from the CD163 expression pattern observed in monocytes and monocyte-derived macrophages from other mammalian species (69). While it is interesting to speculate that altered CD163 expression in mice may be an evolutionary adaptation to reduce arterivirus-induced disease, this theory remains untested. Tissue-, age-, sex-, and species-specific aspects of receptor expression—along with proteolytic “shedding” of receptors (e.g., CD163) from the cell surface—may each play an important role in determining the tropism and disease induced by arteriviruses (77). Finally, it is likely that receptor display only partially explains cellular susceptibility to arterivirus infection. Other host factors that are either required for post-entry replication (host dependency factors) or act to restrict viral replication (restriction factors) may also play an important role, as evidenced by the fact that human macrophages do not support SHFV infection despite their display of receptors fully capable of mediating SHFV entry (68).

### Dichotomous outcomes of arterivirus infection: acute disease versus sub-clinical persistence

**Fig 3:**
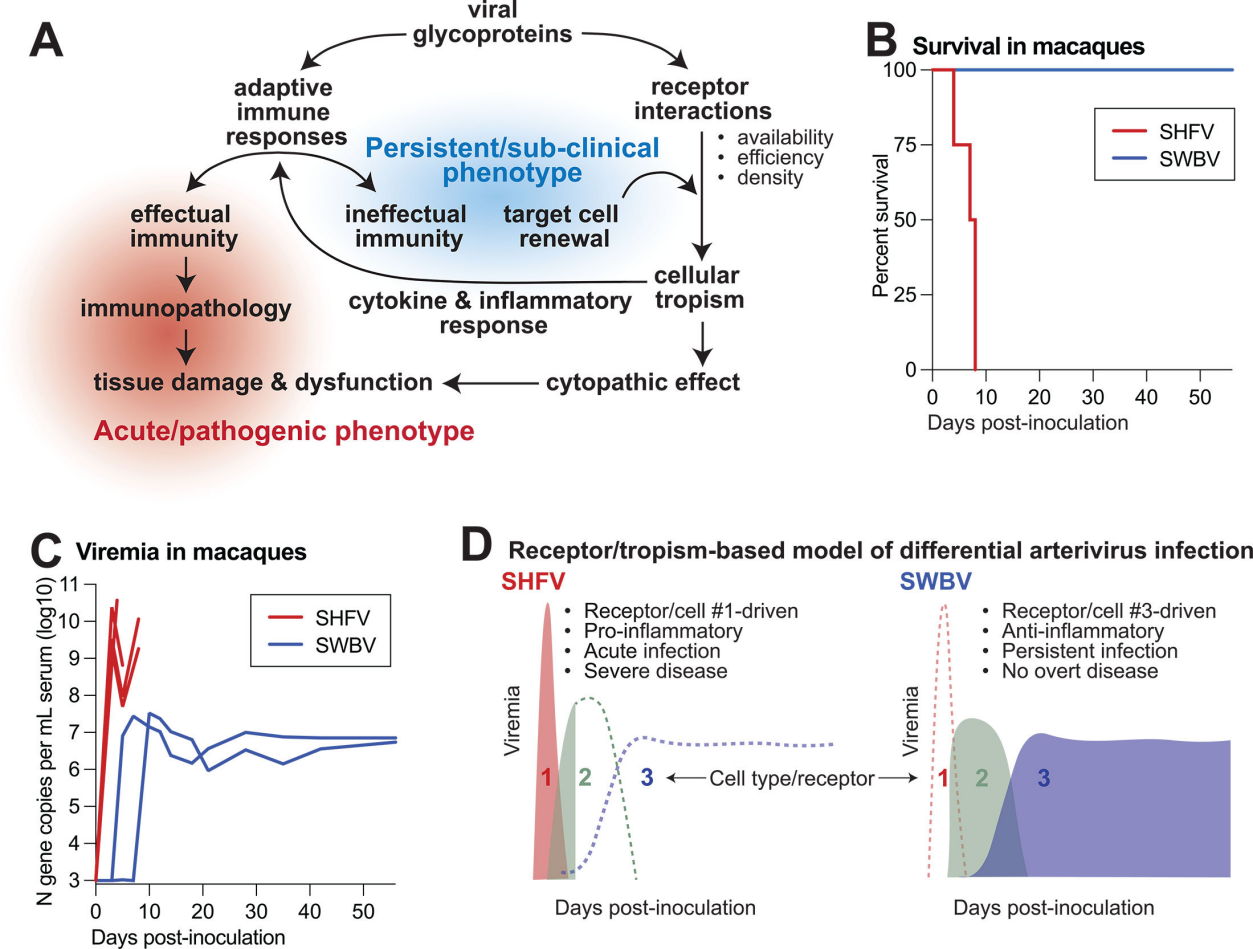
Proposed model of how arterivirus receptor utilization might impact disease. (**A**) Schematic showing major aspects of arterivirus infection and disease, with the persistent/sub-clinical infection phenotype often seen in “natural hosts” shown in blue in contrast to the acute/pathogenic infection phenotype shown in red (7, 16). (**B**) Survival analysis of macaques infected with the disease-causing simian hemorrhagic fever virus (red) versus the Southwest baboon arterivirus (SWBV) that causes a sub-clinical infection (blue). (**C**) Viremia following infection for the same viruses. (**D**) Infection kinetics of SHFV versus SWBV, explained by hypothetical differences in receptor utilization and tropism. Numbers represent distinct cellular compartments rendered susceptible or non-susceptible by virus-specific receptor interactions.

Currently, the mechanism underlying the vastly different diseases produced by different arteriviruses in different hosts is unclear. While there is clearly a pattern of arteriviruses causing persistent infection and sub-clinical disease in “natural hosts” versus acute infection and severe disease in “non-natural hosts,” this dichotomy is not without exceptions. For example, patas monkeys—a presumed natural host of SHFV—do not develop severe disease when experimentally infected with SHFV (78–81). However, infection of macaque monkeys with SHFV is uniformly lethal (14, 16). In fact, macaques develop severe disease when infected with several divergent simian arteriviruses (14, 82), but show no signs of disease when infected with the baboon arterivirus SWBV (7), which instead establishes a persistent infection (Fig. 3). Thus, arterivirus persistence and disease cannot be completely explained by host-specific susceptibility features, nor can they be solely attributed to virus-intrinsic virulence.

One possible explanation for these observations could be that the pattern of receptor utilization (or host immune restriction) in these different scenarios affords these different viruses access to different cellular compartments. In turn, the cellular compartments infected by these viruses could play an important role in shaping the subsequent immune response. Thus, it stands to reason that these viruses (i) infect different cell types and/or (ii) elicit very different immune responses (and corresponding levels of immunopathology), which are also probably influenced by the cell type(s) targeted for infection. More clearly delineating the cellular populations that are infected by arteriviruses, the receptors (e.g., FcRn, Siglec, and CD163) that are used to infect these cells, and the innate factors that restrict arterivirus infection will help build a more comprehensive understanding of arterivirus pathogenesis. Importantly, studies of individual arteriviruses in isolation or in only a single host species are unlikely to reveal the broader mechanisms by which arteriviruses cause disease and/or persist.

### Mechanism of arterivirus persistence

Several arteriviruses cause life-long infections in their animal host. While EAV continuously replicates in an immune-privileged site (the male reproductive tract of stallions), other arteriviruses can be consistently detected at high levels in the bloodstream of their host—a remarkable feat that is unusual among +ssRNA viruses. How arteriviruses accomplish this remains unknown. Arteriviruses have no known latency mechanism, indicating that active replication is required to perpetuate infection. Immune responses are generated against arterivirus glycoproteins, but arteriviruses escape these responses by utilizing “decoy epitopes” that act to attract antibody responses and then rapidly mutate to avoid neutralization, presumably without impacting adjacent receptor-binding domains (RBDs) (7, 83–90). Though other viruses utilize similar strategies to shield their structurally constrained RBDs from direct antibody attack, it is currently unclear what makes the arterivirus version of this strategy so effective. In fact, our recent work has shown that the entire adaptive immune system has a negligible impact on LDV viremia, highlighting the extensive immune-evasion capabilities of these viruses (91).

Many persistent viruses infect cells of the immune system, and this is also true for arteriviruses. However, how persistent arterivirus infection impacts immune function remains largely unknown. LDV infection has been shown to cause minor changes to the architecture of secondary lymphoid organs (92–96), but the persistent elevation of lactate dehydrogenase (LDH) in LDV-infected mice remains one of the few indications—other than the measurement of the virus itself—that host immune homeostasis is abnormally altered. Elevated serum LDH is thought to stem from reduced LDH clearance capacity of the body-wide monophagocytic system; however, the anatomic location, responsible cell type(s), and reason for impaired clearance have never been elucidated. As such, the impact of persistent arterivirus infection on chronic processes like aging, obesity, autoimmunity, cancer, or co-infections remains largely unexplored.

### Evolutionary implications of arterivirus receptors and glycoproteins

**Fig 4:**
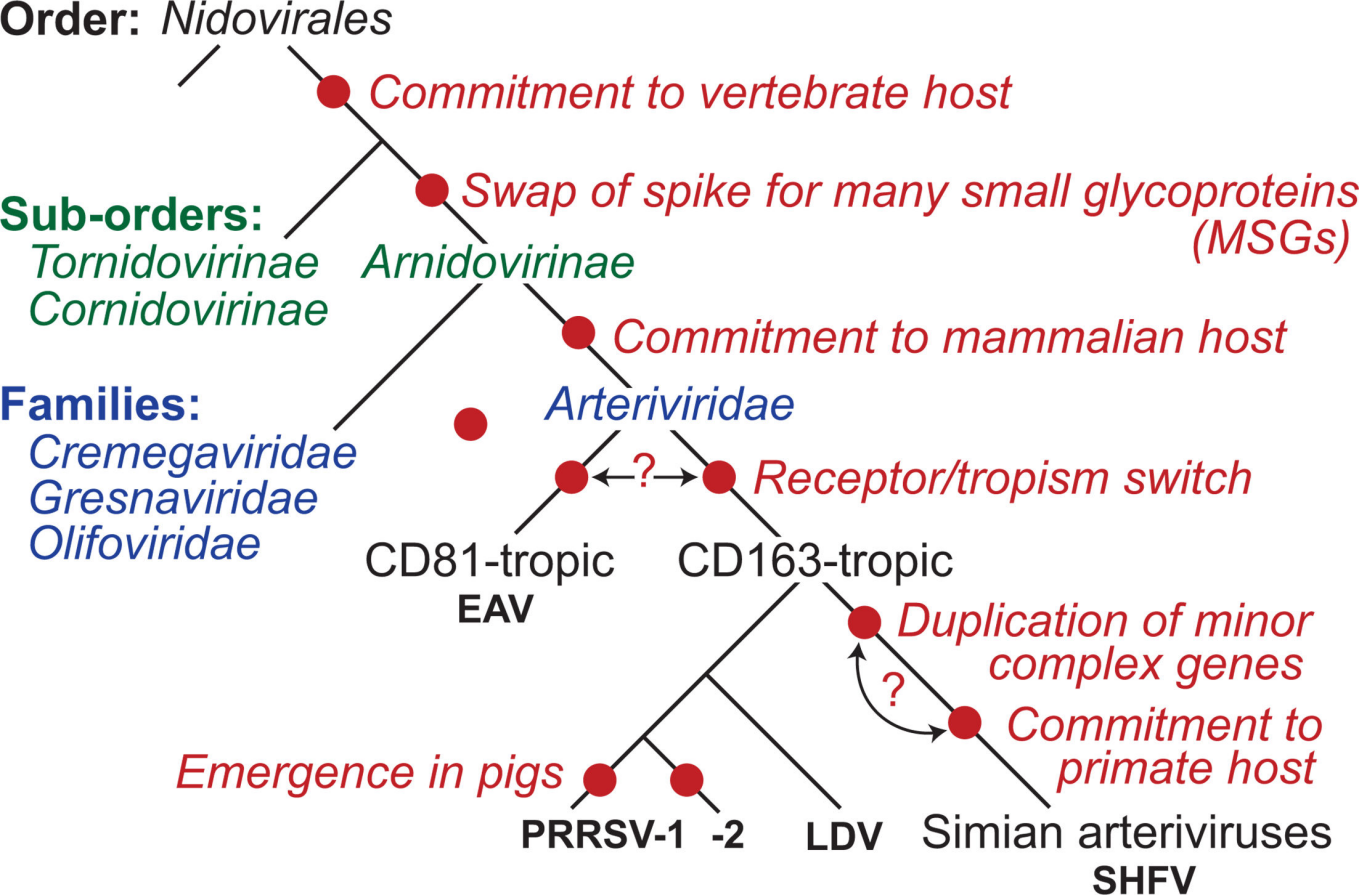
Important events in the evolutionary history of arteriviruses.

The *Arteriviridae* are a taxonomic family within the larger Order *Nidovirales* (Fig. 4). Currently, all viruses within *Arteriviridae* infect mammals and share the previously described arrangement of multiple small glycoprotein (MSG) genes, presumptively organized into minor and major complexes. Several genetically-related viruses discovered in reptiles contain putative glycoprotein genes arranged in a similar manner (3), but these are currently classified into their own Families (*Cremegaviridae*, *Gresnaviridae*, and *Olifoviridae*) that, together with the *Arteriviridae,* comprise the *Arnidovirinae* sub-Order. Thus, the MSG paradigm defines the *Arnidovirinae* sub-Order and stands in stark contrast to the “single spike glycoprotein” arrangement that is common among nidoviruses from phylogenetically adjacent sub-Orders (*Tornidovirinae* and *Cornidovirinae*, which contain the coronaviruses). A large number of divergent nidoviruses have recently been described in non-chordate hosts (Phyla: *Arthropoda*, *Annelida*, *Cnidaria*, *Mollusca*, *Nematoda*, and *Platyhelminthes*), potentially implying a deep evolutionary relationship between nidoviruses and non-vertebrate hosts (4). Although the glycoprotein genes of these “non-vertebrate” nidoviruses deserve further scrutiny, there are clear examples of these viruses encoding a single spike glycoprotein gene (e.g., the *Mesoniviridae*). Given that a single replacement event (i.e., spike replacing MSG vs MSG replacing spike) is the most parsimonious explanation for the current distribution of Nidovirus glycoproteins, it seems likely that the common ancestor of vertebrate-infecting nidoviruses likely possessed a single spike glycoprotein. This also implies that an ancestral arterivirus exchanged spike in favor of the MSG arrangement. The advantages of an MSG arrangement compared to spike are not clear, but may be related to immune evasion and persistence, as MSGs elicit poorly neutralizing antibodies against linear epitopes, whereas spike glycoproteins are highly immunogenic and elicit strong neutralizing antibodies against conformational epitopes (97, 98). Within the *Arteriviridae*, there also now appears to be a clear connection between receptor utilization and tropism, with CD163-tropic viruses infecting macrophages and a CD81-tropic virus (i.e., EAV) that exhibits broader cellular/tissue tropism (72, 99). This dichotomy clearly indicates that a receptor switch took place at some point in the evolutionary history of arteriviruses. While the directionality of this switch is currently unknown, understanding the receptor(s) used by further divergent arteriviruses like wobbly possum disease virus (WPDV)—which itself has a unique tropism among arteriviruses—will be illuminating (100).

### Simian arteriviruses

The advent of unbiased metagenomic sequencing revealed that divergent arteriviruses infect wild monkeys throughout Africa. Despite many of these “simian arteriviruses” or “simarteviruses” sharing only ~50% nucleotide identity with one another, they form a monophyletic clade and share a duplication of the minor complex genes (GP2′/3′/4′ and E′), bringing the total putative number of unique structural genes to 11. These unique genetic features, along with the broad geographic distribution and species diversity of infected monkeys, suggest that simian arterivirus infection in wild monkeys is ancient. Simian arteriviruses also display striking levels of genetic diversity within primate communities and within persistently infected individuals.

**Fig 5:**
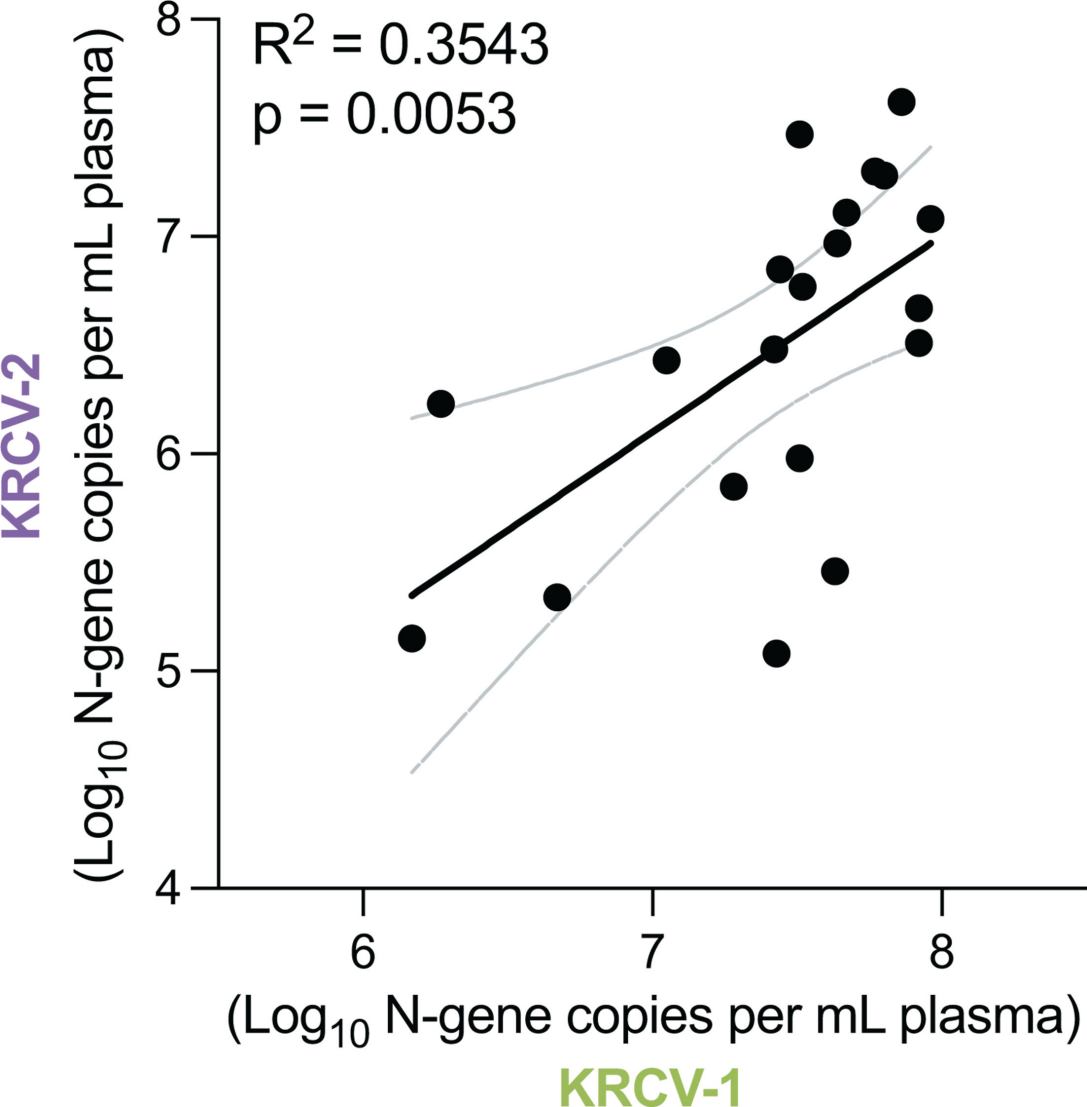
Red colobus arteriviruses do not exhibit niche exclusion. Plasma viral loads from red colobus monkeys co-infected with two divergent arteriviruses, Kibale red colobus virus (KRCV)-1 and KRCV-2 (90). If the two viruses competed with one another for the same target cells (i.e., “niche”), their viral loads would be inversely proportional. Instead, the opposite is observed (solid black line shows simple linear regression with dashed gray line showing the 95% confidence interval).

With the exception of SHFV, the biology of the simian arteriviruses remains almost completely unexplored. These viruses do not productively infect immortalized cell lines like MA-104 cells, though simian arteriviruses have recently been cultured in primary macaque splenocytes, macaque iPSC-derived macrophages, or mice engrafted with macaque hematopoietic stem cells (101). Overexpression of CD163 and FcRn also permits infection of MA-104 cells with certain simian arteriviruses (71), perhaps indicative of inefficient receptor interactions that can be overcome with more favorable stoichiometry. Notably, none of these host systems universally support simian arterivirus replication, implying that these viruses require significantly different host factors for productive infection. A striking natural example can be found in the red colobus monkeys of Kibale National Park in Uganda, many of whom are persistently infected with two highly divergent simian arteriviruses (KRCV-1 and KRCV-2). These viruses not only co-circulate within the same community and individuals, but do not appear to compete with one another (90) (Fig. 5). This suggests that KRCV-1 and KRCV-2 occupy distinct niches within the red colobus host—most likely unique cell types that are exclusively infected by KRCV-1 or KRCV-2 due to the use of different receptors by these different viruses.

The relatively limited sampling of African primates suggests many more highly divergent simian arteriviruses remain to be discovered. Given the shared evolutionary history between humans and other primates and the fact that many human pathogens have non-human-primate origins, understanding simian arterivirus biology in greater detail seems prudent. Generally, receptor expression is a major determinant of cellular susceptibility to viral infection (102, 103) and, so far, this also appears to be true for arteriviruses. Thus, characterizing the host receptors utilized by these viruses may be of significant value for risk-stratifying the zoonotic potential of specific simian arteriviruses (15). The function of the “prime” complex—and why it is so important for infection of primates—may be a particularly important piece in this puzzle.

## OUTLOOK AND PRACTICAL CONSIDERATIONS

### Understanding arterivirus entry for improved medical countermeasures

The fact that arteriviruses can persistently infect immunocompetent adult animals suggests that immune-derived countermeasures—that is, vaccines and monoclonal antibodies—are poised to fail against arteriviruses. Indeed, current PRRSV vaccines can only provide modest protection against homologous strains (97, 98, 104). However, it is worth noting that PRRSV vaccines have been developed empirically, without a clear understanding of the molecular interactions that govern arterivirus entry. Given that many effective vaccines elicit antibodies that target receptor-binding domains on viral glycoproteins, a more detailed understanding of the interactions between arterivirus glycoproteins and their host receptor(s) should enable the rational design and development of countermeasures that are more effective at blocking these interactions.

### Prediction of the zoonotic threat posed by arteriviruses

Given the mammals that are currently known to be infected by arteriviruses, it seems unlikely that humans are intrinsically resistant to this entire family of viruses. Recent studies have shown that SHFV can infect human cells; however, the potential of arteriviruses to infect humans remains unclear (68, 105). Receptor utilization is an important determinant of viral cross-species infection in general, and this also appears to be true for the arteriviruses. However, the viral complexes, proteins, domains, and residues that govern these interactions remain poorly defined. Mapping key virus:receptor interactions is critical towards developing a set of rules for explaining arterivirus behavior at the molecular level, which will aid in the risk-stratification of arteriviruses based on molecular compatibility with potential hosts, similar to what is currently done for influenza.

### Understanding points of host vulnerability to viral infection

Many of the host factors that arteriviruses use to enter cells have been identified as critical receptors for other viruses: hepatitis C virus (HCV) requires binding to CD81 at the same host interface as EAV (72, 106); African swine fever virus (ASFV) uses CD163 for entry (107); and FcRn has recently been described as a receptor for echoviruses and astroviruses (108–111). These findings suggest that unrelated viruses have converged upon the same host factors to successfully infect mammalian cells, raising intriguing questions about specific points of host vulnerability to viral hijacking. Thus, host-directed countermeasures may be a fruitful strategy that may aid in overcoming some of the aforementioned challenges in developing anti-arteriviral countermeasures. (Box 1).

Box 1. **Outstanding questions in arterivirus entry**

- Are additional host molecules beyond CD163, CD81, and FcRn important for arterivirus entry?
- How does receptor utilization impact infection dynamics?
  - How does receptor/co-receptor utilization vary across arteriviruses and in different hosts?
  - How does receptor/co-receptor utilization relate to cellular tropism and disease?
  - Does receptor/co-receptor utilization change over the course of chronic arterivirus infection?
- What viral glycoproteins interact with CD81 and FcRn?
- How are glycoprotein/receptor interactions coordinated during viral entry?
  - Is receptor-binding step-wise, cooperative, or simultaneous?
  - What are the locations of receptor engagement (e.g., cell surface, early endosome, and late endosome)?
- Which glycoprotein acts as the viral “fusion machine”?
- How do differences in receptor orthologs act as barriers to cross-species infection?
- What advantages/trade-offs are conferred by arterivirus glycoproteins, compared to the ancestral nidoviral “spike” glycoprotein?
- What additional post-entry factors impact arterivirus tropism?
